# Associating Emergency Medical Services personnel’s workload, trauma exposure, and health with the cortisol, endocannabinoid, and *N*-acylethanolamine concentrations in their hair

**DOI:** 10.1038/s41598-020-79859-x

**Published:** 2020-12-29

**Authors:** Alexander Behnke, Alexander Karabatsiakis, Aniko Krumbholz, Sarah Karrasch, Gustav Schelling, Iris-Tatjana Kolassa, Roberto Rojas

**Affiliations:** 1 Clinical and Biological Psychology, Institute of Psychology and Education, Ulm University, 89081 Ulm, Germany; 2 Institute of Psychology, University of Innsbruck, 6020 Innsbruck, Austria; 3 Institute of Doping Analysis and Sports Biochemistry (IDAS) Dresden, 01731 Kreischa, Germany; 4 Department of Anaesthesiology, Ludwig Maximilians University, 82131 Munich, Germany; 5 University Psychotherapeutic Outpatient Clinic, Institute of Psychology and Education, Ulm University, 89073 Ulm, Germany

**Keywords:** Steroid hormones, Neuroendocrinology, Psychology, Biomarkers, Endocrinology, Health occupations

## Abstract

In their line of duty, Emergency Medical Services (EMS) personnel are exposed to chronically stressful working conditions and recurrent traumatic events, which increase their risk for detrimental health outcomes. Here, we investigated whether this risk is due to altered regulation of the hypothalamus–pituitary–adrenal (HPA) axis and the endocannabinoid system. Therefore, 1 cm hair strands were collected from a cohort of 72 German EMS personnel in order to measure concentrations of cortisol, endocannabinoids [i.e., anandamide (AEA), 2-arachidonoylglycerol (2-AG)], and *N*-acylethanolamines [i.e., stearoylethanolamide (SEA), oleoylethanolamide (OEA), and palmitoylethanolamide (PEA)]. Rank correlation analyses were conducted to test associations of cortisol, endocannabinoid, and *N*-acylethanolamine concentrations with the EMS personnel’s workload, lifetime trauma exposure, and mental and physical health problems. We found a negative correlation between cortisol and 2-AG concentrations in hair. Higher hair cortisol was associated with higher workload. Reported traumatic stress during childhood and later in life as well as more severe depressive and physical stress symptoms were associated with elevated 2-AG, SEA, OEA, and PEA concentrations. Future longitudinal research needs to address the prospect of tracing biomolecular markers of glucocorticoid, endocannabinoid, and *N*-acylethanolamine activity as a predicting value of the long-term course of mental and physical well-being.

## Introduction

Emergency Medical Services (EMS) personnel play an essential role in our society. However, due to their work nature, they are constantly exposed to mental and physical stressors, i.e. (i) chronic stress due to adverse work conditions such as shift work including regular night shifts, as well as (ii) recurrent encounters of potentially traumatic events at mission sites^1–3^. As a result, EMS personnel show an increased prevalence of stress- and trauma-related mental as well as physical health problems, including posttraumatic stress disorder (PTSD), major depressive disorder (MDD), and concomitant physical ailments^1,3–5^. Due to EMS personnel’s burden of stress and health risks, it is highly relevant to investigate how their exposure to mental stress and trauma gets embedded in biological systems and elevates their vulnerability for negative health conditions. However, the biological processes that translate recurrent exposure to chronic and traumatic stress into detrimental health effects are not sufficiently understood.

Existing studies indicate, stress-induced changes in the regulation of the body’s endocrine stress-response system could mediate the aetiology of stress-related (psycho-)pathology^6,7^. The hypothalamus–pituitary–adrenal (HPA) axis acts as a main regulator of body’s endocrine stress reaction by triggering the release of glucocorticosteroid hormones from the adrenal glands into the blood stream^8^. The glucocorticosteroid hormone cortisol is probably the most intensively investigated bioactive molecule in the context of stress and trauma-associated psychopathology^9,10^. During phases of acute and chronic stress, cortisol induces glucose release by glycolysis from the liver to various cells and tissues within minutes and persist over hours^8^. Under physiological conditions, increase in cortisol concentrations inhibits further cortisol secretion via a negative feedback loop^8^. In addition, the HPA axis’ negative feedback mechanism is pivotally conditioned by the *endocannabinoids* anandamide (AEA) and 2-arachidonoylglycerol (2-AG)^6,7,11,12^. Endocannabinoids are bioactive signalling lipids derived from arachidonic acid or other polyunsaturated fatty acids that are enzymatically synthesised on demand from cell membranes of the central nervous system, blood cells, and other peripheral tissues^13,14^. According to conceptual models^6,7,11,12^, in the absence of acute stress, the tonic activity of central-nervous AEA regulates the activity of the HPA axis. With exposure to stress, AEA activity decreases and disinhibits the HPA axis, which eventually triggers the secretion of cortisol. Subsequently, the stress-related activity of glucocorticoids stimulates the synthesis and secretion of AEA and 2-AG. The timely delayed increase of 2-AG acts as a turn-off signal to downregulate HPA-axis activity once the stressful situation has ended^11,12^.

Alterations in the mutual endocannabinoid–glucocorticoid regulation could compromise the physiological stress response and this could initiate a vulnerability to develop stress-related health problems. Another important pathway for the development of stress-related health problems could be alterations in the regulation of the immune system. In fact, individuals with chronic ongoing stress or with history of traumatic experiences exhibit chronic low-grade inflammatory activity^15–17^, which has been consistently linked to depressive and posttraumatic stress symptoms^17–19^. Persistent changes in HPA-axis activity might contribute to an altered immune regulation, as glucocorticoids exert immunosuppressive and anti-inflammatory effects^8^. Furthermore, there is consistent evidence that activated immune cells secrete endocannabinoids such as AEA and 2-AG as well as chemically closely related *N*-acylethanolamines (NAEs) such as stearoylethanolamide (SEA), oleoylethanolamide (OEA), and palmitoylethanolamide (PEA)^14^, which inhibit immune cells from further secretion of pro-inflammatory cytokines^13,20^. Therefore, endocannabinoids and NAEs are considered to facilitate the downregulation of the initial immune responses back to baseline^13,20^. Correspondingly, endocannabinoids and NAEs were found to reduce neural toxicity, lower oxidative stress, and attenuate visceral and neuropathic pain^21–23^.

The central role of endocannabinoids, NAEs, and glucocorticoids in regulating the body’s short- and long-term stress adaptation as well as immune activity points towards the relevance to investigate their mutual dependency in the aetiology of stress and trauma-related disorders^6,7,20,24,25^. In this context, stress research has been focusing on measuring the concentrations of glucocorticoids, endocannabinoids, and NAEs in hair samples. Hair concentrations are considered to retrospectively represent the intra-corporeal activity of measured biomolecules in the course of several weeks to months^10,26–28^. Such studies provide evidence for altered hair concentrations of cortisol (HCC), endocannabinoids, and NAEs associated with exposure to chronic and traumatic stress as well as related health problems. First of all, there is meta-analytical evidence of elevated HCC among individuals with ongoing occupational stress (e.g. shift workers, nursing staff)^9^. However, longitudinal studies reported inconsistent prospective associations of intensifying stress and increasing HCC^29,30^. To our best knowledge, endocannabinoids and NAEs have not yet been investigated among chronically stressed professionals. Secondly, studies on traumatic stress have observed an increase in HCC over a period of several weeks to months, followed by a return of HCC to pre-trauma levels or below^31–33^. It is discussed whether the trauma-triggered strong increase of cortisol activity sensitises the HPA-axis feedback loop, and, thereby, leads to a persistently reduced cortisol secretion^34,35^. However, findings on the association between lifetime trauma exposure and reduced HCC are inconsistent^36–38^. Also with regard to traumatic stress in early life, there are inconsistent findings on lowered, unchanged, or heightened HCC among adults who experienced severe abuse or neglect in childhood and adolescence, i.e. childhood maltreatment (CM)^36,39^. Additionally, lowered cortisol activity has been suggested to be a risk factor for the development of trauma-related disorders after re-traumatisation^32,40,41^. Correspondingly, a meta-analysis^9^ indicated a marginal negative association between HCC and the severity of posttraumatic stress symptoms, whereas no consistent association was found with depressive symptoms.

Compared to research on HCC alterations, limited research has been conducted on alterations in hair endocannabinoid and NAE concentrations in the context of stress and health. Among heavily traumatised survivors of the Ugandan civil war^42^, higher lifetime trauma exposure and more severe PTSD symptoms were associated with lower levels of PEA, SEA, and OEA in hair. In a study among European women in one month postpartum^43^, CM exposure was associated with higher 1-AG and lower SEA concentrations in hair. Women with a lifetime diagnosis of one or more psychiatric disorders also showed reduced OEA, SEA, and PEA concentrations in hair^43^.

To advance the knowledge on the role of the glucocorticoid, endocannabinoid, and NAE system in the development of stress-related health problems, we aimed to investigate possible alterations in the mutual regulation of cortisol as well as selected endocannabinoids and NAEs in a chronically stressed and trauma exposed cohort of EMS personnel. Through this study, we anticipated to find (i) increased HCC among EMS personnel with higher quantitative workload, and (ii) HCC to be associated with the personnel’s lifetime exposure to potentially traumatic events experienced either in private life or in the line of duty. (iii) We explored the association between HCC and CM exposure as well as (iv) the associations between HCC and the severity of posttraumatic, depressive, and physical stress symptoms. (v) Furthermore, we explored the associations between the concentrations of endocannabinoids and NAEs in the EMS personnel’s hair and their workload, lifetime trauma exposure, and stress-related symptoms. (vi) In line with aforementioned conceptual models^6,7,11,12,28^, we also expected to find negative correlations of cortisol with AEA and 2-AG concentrations in hair.

## Material and methods

### Recruitment and characterisation of the study cohort

All study procedures were approved by the Ulm University ethics committee. All study procedures and methods were performed in accordance with the Declaration of Helsinki. Analyses were based on a cohort of *N* = 72 Emergency Medical Technicians stationed at two German Red Cross ambulance stations located in Ulm and Heidenheim (State of Baden-Württemberg, Germany). For a detailed summary of the study cohort’s sociodemographic and lifestyle-related characteristics see Table 1. Recruitment took place after regular on-the-job education events. Individuals interested in participation received an invitation email with access to an online survey. After participants were provided with detailed information on general study aims and its procedures, a written informed consent was obtained from them. Afterwards, participants answered questionnaires on their mental and physical health status, workload, and lifetime trauma exposure.

Out of 318 EMS employees working at the two ambulance stations, 115 of them (36.2%) participated in the study. Among them, a subgroup of 97 participants agreed to provide hair strands for the analysis of steroid and lipid concentrations in hair. Due to relatively short hair length especially among men, we decided to collect hair strands with a minimum of 1 cm length. Hair samples of 94 participants were preprocessed to a standardised segment length of 1 cm and shipped to the laboratory, whereas three samples of hair shorter than 1 cm were excluded. Due to analytical limitations, steroids and lipids could not be measured in hair samples of less than 2 mg, resulting in the exclusion of another two hair samples. Data of *n* = 20 participants were excluded due to a priori defined pathophysiological conditions that were reported to systemically confound HCC^9,44^, i.e. chronic inflammatory diseases (*n* = 1 Morbus Crohn), chronic metabolic diseases (*n* = 4 hypothyroidism; *n* = 1 type-II diabetes; *n* = 1 hyperinsulinemia; *n* = 1 polycystic ovarian syndrome; *n* = 1 chronic renal problems), rare blood diseases (*n* = 1 thalassemia; *n* = 1 haemolysis; *n* = 1 hereditary factor-X deficiency), regular cardiovascular medication (*n* = 6 anti-noradrenergic drugs), regular psychiatric medication (*n* = 1 antiepileptica), multiple-substance consume disorder (*n* = 1 cannabis/alcohol/cocaine consumption disorder). Biological variables could be measured in a varying number of the 72 available hair samples, i.e. cortisol: *n* = 53; 2-AG: *n* = 60; AEA: *n* = 31; SEA: *n* = 72; PEA: *n* = 72; OEA: *n* = 72. To maximise statistical power, subsequent analyses were computed with all available data.

### Clinical assessment

Posttraumatic stress symptoms regarding an index event were assessed using the German PTSD Checklist for DSM-5 (PCL-5)^45^. On 20 items, participants reported the intensity to which they felt impaired by intrusions, avoidance, hyperarousal, as well as negative alterations in mood and cognition in the previous month. Responses were recorded on a 5-point Likert scale from 0 (“*not at all”*) to 4 (“*very strong”*), and aggregated to a sum score (Cronbach’s α = 0.92). On the 9-item depression scale of the German Patient Health Questionnaire (PHQ-9)^46^, participants indicated the severity of nine depressive symptom types during the past two weeks. Responses were recorded on a 4-point Likert scale from 0 (“*not at all*”) to 3 (“*almost every day*”), and aggregated to a sum score (Cronbach’s α = 0.81). Physical stress symptoms were assessed using the physical symptom scale of the German Patient Health Questionnaire (PHQ-15)^46^. On 15 items (including two PHQ-9 items for disturbed sleep), participants reported the extent to which they felt impaired by e.g. headache, back pain, stomach aches or sleep disturbances in the previous month. Responses were recorded on a 3-point Likert scale from 0 (“*not at all”*) to 2 (“*very strong”*), and aggregated to a sum score (Cronbach’s α = 0.84). According to the questionnaires’ cut-off values to screen for clinically relevant symptoms, the present cohort reported weak posttraumatic and depressive as well as weak to moderate physical stress symptoms (Table 1).

**Table 1:** Sample characteristics.

	*N*	*Med* (*IQR*) or frequency	Empirical range	Theoretical scale range
Quantitative workload	62	0.07 (0.80)	[− 1.50, 1.90]	*z*-scaled
Childhood maltreatment exposure (MACE-20)	61	6.66 (10.00)	[0.00, 55.00]	[0.00, 100.00]
Number of experienced major life events (private life) (LEC-5)	62	4.00 (4.30)	[0.00, 13.00]	[0.00, 34.00]
Number of experienced potentially traumatic mission aspects (RESQ-CE)	61	5.00 (4.00)	[1.00, 10.00]	[0.00, 10.00]
Severity of posttraumatic stress symptoms (PCL-5)	60	5.00 (11.00)	[0.00, 39.00]	[0.00, 80.00]
Severity of physical symptoms (PHQ-15)	59	5.00 (7.00)	[0.00, 21.00]	[0.00, 30.00]
Severity of depressive symptoms (PHQ-9)	59	4.00 (6.00)	[0.00, 17.00]	[0.00, 27.00]
Age (in years)	62	25.00 (14.00)	[18.00, 54.00]	
EMS work experience (in years)	62	3.08 (5.0)	[0.00, 33.00]	
Sex	62	Women: 27 (43.5%) Men: 35 (56.5%)		
Body Mass Index (BMI) ^a^	24	25.95 (6.30)	[20.00, 37.20]	
Sports exercises (hours per week)	71	2.00 (3.75)	[0.00, 14.00]	
Frequency of hair washing per week	71	7.00 (3.00)	[3.00, 14.00]	
Hair treatments ^b^	71	No: 63 (87.5%) Yes: 8 (11.3%)		
Acute infections ^c^	72	No: 43 (59.7%) Yes: 29 (40.3%)		
Natural hair colour ^d^	71	Red/blond: 30 (42.3%) Brunet/black: 41 (57.7%)		
Major stress events within four weeks before study participation ^e^	59	No: 42 (71.2%) Yes: 17 (28.8%)		

*Med *median, *IQR *interquartile range.

^a^BMI was introduced to the questionnaires after study start.

^b^Bleaching, dyeing, tinting, and permanent waving in the last 6 weeks before hair sample collection were considered as hair treatment.

^c^Self-reported common colds, flu, influenza, angina, and cystitis at the time of hair sample collection or within four weeks before.

^d^Only a small percentage of participants had red (*n* = 2) or black (*n* = 3) as natural hair colour. Participants with grey hair were categorised according to their initial natural hair colour.

^e^See Supplementary Table S5 for further details.

Three questionnaires were employed to measure lifetime trauma exposure. On the 20-item self-report version of the German Maltreatment and Abuse Chronology of Exposure scale (MACE-20)^47^, participants indicated with *yes* or *no* which forms of maltreatment (i.e. emotional and physical neglect and emotional, physical or sexual abuse by parents or siblings as well as witnessed violence among parents or siblings) they experienced during their childhood and adolescence. All answers were summarised to an overall score representing the total childhood maltreatment exposure on a scale from 0 to 100. In this study cohort, most participants reported no to mild CM exposure while a minority was exposed to mild to moderate levels of CM. The MACE-20 assesses the total exposure to childhood maltreatment but does not differentiate which forms of maltreatment were experienced at an age. The German Life Event Checklist for DSM-5 (LEC-5)^48^ was used to quantify the exposure to potentially traumatic events in adulthood. Participants indicated which of 31 types of major life events they experienced or witnessed, including natural disasters and human-made violence or incidents, but also severe illness or death. We excluded the answering option of being confronted on duty and instructed the participants to focus on their private life only. Work-related potentially traumatic events were assessed using the Rescue and Emergency Situations Questionnaire (RESQ)^2^. Participants indicated which of 31 emotionally burdensome aspects of emergency medical rescue mission they experienced, including ten potentially traumatising mission aspects. In further analyses, we used the number of experienced potentially traumatic mission aspects (RESQ-CE score).

To objectify the current quantitative workload participants reported the number of nightshifts, medical rescue operations, and routine patient transports they carried out in the previous month prior to the assessment. Reported quantities were *z*-standardised and averaged to the variable “quantitative workload”. In addition, participants reported their age, sex, work experience, and experience of major life events within the last month. While hair sampling, participants reported their natural hair colour as well as the frequency and type of hair treatments (e.g. bleaching, colouring, dyeing, permanent waving, and washing frequency). Additionally, they indicated the frequency of sports exercises, chronic diseases, or acute infections, as well as consumption of medication and drugs.

### Sampling and pre-processing of hair strands

Trained academic staff used laboratory gloves to collect three hair strands from a posterior vertex position of the scalp avoiding the hair follicle. Majority of participants, especially middle-aged men had short and thin scalp hair (minimal length approx. 5 mm); hence, available hair was limited in length and weight. Following a standardised protocol, hair strands were cut to 1 cm segments, weighed (hair samples with minimal 1 cm length: *n* = 94, *Med* = 7.50 mg, *IQR* = 6.13 mg, range: 1.70–50.90 mg), and stored into sterile Eppendorf tubes.

### Laboratory analyses of steroid and lipid concentrations in hair

Hair analysis was performed using a validated protocol^28^. Due to the relatively low hair-sample quantities, technical adaptations were necessary. Hair samples were powdered using a ball mill (Homogenizer FastPrep-24, MP Biomedicals, USA). After adding internal standards (cortisol-d4, AEA-d4, PEA-d4, 2AG-d5), extraction with 1.5 mL methanol was carried out in an ultrasonic bath at 50 °C for 6 h. The liquid phase was separated and cleaned by solid-phase extraction (SPE). Hair residues from methanol extraction were hydrolysed with 1.0 mL 0.5 N KOH-solution for 18 h at 60 °C. After separating the liquid phase, the clean-up was performed by SPE. Extracts from both SPE-runs were combined, evaporated, and reconstituted with HPLC-buffer. Hair concentrations were measured by HPLC-HR-MS/MS-technology using the Agilent HPLC system 1290 infinity and the Sciex TripleTOF 6600 mass spectrometer. Chromatographic separation was operated on an Agilent ZORBAX-Eclipse XDB-C8-column using a linear gradient using the buffer A (water/acetonitrile (95:5), 2 mmol NH_4_AC, 0.1% acetic acid) and buffer B (water/acetonitrile (5:95), 2 mmol NH_4_Ac, 0.1% acetic acid)^28^.

### Statistical analyses

Statistical analyses were performed with R^49^. Nonparametric analyses were conducted as most of the variables were not normally distributed. Using Spearman rank correlations and Mann–Whitney *U*-tests, the steroid and lipid concentrations in hair were investigated for potential influences of age, sex, natural hair colour, hair treatments, frequency of hair washing, body mass index (BMI), experience of major life events within the last 31 days, infections within in the last month, also frequency and duration of sports exercises^9,44,50^. Correlations between the steroid and lipid concentrations in hair as well as their associations with stress, trauma, and health variables were analysed using Spearman rank correlations. In the case of significant associations of the steroid and lipid concentrations in hair with covariates, these associations were statistically controlled using semi-partial Spearman rank correlations (using the R package *ppcor*^51^).

## Results

### Potentially confounding influences

Group comparisons indicated higher concentrations of OEA, PEA, and (in trend) SEA among women than men (Supplementary Table S1) as well as among participants who applied cosmetic hair treatments within six weeks before hair sampling (Supplementary Table S2). Biological hair parameters did not differ depending on participants’ acute inflammatory states, natural hair colour, or recently experienced major stress events (Supplementary Tables S3–S5). Spearman correlations (Supplementary Table S6) indicated lower PEA concentrations in the hair of older participants. Cortisol, endocannabinoid, and NAE hair concentrations were not associated with the frequency of hair-washing, sports exercises, or the BMI.

### Associations of cortisol, endocannabinoid, and NAE hair concentrations with stress exposure and stress-related symptoms

Table 2 displays the results of bivariate and semi-partial Spearman correlation analyses. Semi-partial correlations were computed to control SEA and OEA concentrations for influences of sex and hair treatment as well as PEA concentrations for influences of sex, age, and hair treatment (see Supplementary Table S7 for zero-order correlations). EMS personnel with higher quantitative workload showed higher HCC. However, HCC was not linked to CM exposure, exposure to potentially traumatic events in private life or on duty, or the severity of mental and physical stress symptoms.

**Table 2:** Results of bivariate and semi-partial correlation analyses.

	Cortisol	2-AG	AEA	SEA^1^	OEA^1^	PEA^2^
**Quantitative workload**						
*r*_S_	0.419**	− 0.122	− 0.202	− **0**.186*	0.046	− 0.016
*p*	0.005^a^	0.387^e^	0.303^i^	0.043^l^	0.618^l^	0.827^l^
**Childhood maltreatment exposure (MACE-20 score)**						
*r*_S_	− 0.023	0.290*	0.086	0.215*	0.286**	0.235**
*p*	0.886^b^	0.039^f^	0.669^j^	0.019^l^	0.002^l^	0.002^l^
**Number of experienced major life events (private life) (LEC-5 score)**						
*r*_S_	− 0.169	− 0.018	0.133	0.027	0.237**	0.175*
*p*	0.274^a^	0.900^e^	0.499^i^	0.771^l^	0.009^l^	0.019^l^
**Number of experienced potentially traumatic events on duty (RESQ-CE score)**						
*r*_S_	− 0129	− 0.052	0.091	− 0.045	0.080	− 0.026
*p*	0.408^b^	− 0.716^f^	0.652^j^	0.628^l^	0.385^l^	0.731^l^
**Severity of posttraumatic stress symptoms (PCL-5 score)**						
*r*_S_	− 0.027	0.097	− 0.008	0.143	0.109	0.056
*p*	0.864^c^	0.503^g^	0.969^k^	0.125^m^	0.242^m^	0.458^m^
**Severity of physical symptoms (PHQ-15 score)**						
*r*_S_	− 0.249	− 0.087	− 0.063	0.110	0.373***	0.204**
*p*	0.116^d^	0.551^h^	0.760^k^	0.241^n^	< 0.001^n^	0.007^n^
**Severity of depressive symptoms (PHQ-9 score)**						
*r*_S_	− 0.208	− 0.103	− 0.050	0.279**	**0**.197*	0.079
*p*	0.192^d^	0.479^h^	0.809^k^	0.002^n^	0.035^n^	0.299^n^

*p < 0.050, **p < 0.010, ***p < 0.001, two-tailed. Bold-faced correlation are significant. Underlined *p*-values are significant after correction with false discovery rate. Semi-partial Spearman correlations were computed to control the associations of ^1^sex and hair treatment or ^2^sex, age, and hair treatment with the respective hair concentration. Sample sizes varied due to differing number of detected steroids and lipids in hair, as well as due to missing values in sociodemographic and psychological data (for details see the methods section). Each correlation was analysed using all respectively available data of the whole study cohort: *n* = ^a^44, ^b^43, ^c^42, ^d^41, ^e^52, ^f^51, ^g^50, ^h^49, ^i^28, ^j^27, ^k^26, ^l^60, ^m^59, ^n^58.

Regarding endocannabinoids and NAEs, higher quantitative workload correlated with lower SEA, but not with any other investigated lipid. CM exposure was associated with higher concentrations of 2-AG, SEA, OEA, and PEA. Participants who reported exposure to more potentially traumatic events in private life had higher OEA and PEA hair concentrations. There were no significant associations of endocannabinoid or NAE hair concentrations with exposure to potentially traumatic mission aspects. Hair concentrations of endocannabinoids and NAEs did not consistently correlate with the participants’ subclinical posttraumatic, depressive, and physical stress symptoms. Higher OEA and PEA concentrations were associated with more severe subclinical physical symptoms; higher SEA and OEA concentrations were linked to more severe subclinical depressive symptoms.

### Associations between cortisol, endocannabinoid, and NAE hair concentrations

Correlation analyses (Table 3) indicated a negative association of cortisol and 2-AG concentrations in hair. Hair concentrations of 2-AG, SEA, OEA, and PEA were positively correlated, except for the insignificant association of 2-AG and OEA concentrations. AEA was not related to the concentrations of cortisol, 2-AG, SEA, OEA, PEA in hair.

**Table 3:** Correlations and descriptive statistics of cortisol and endocannabinoid concentrations in hair.

	Cortisol	2-AG	AEA	SEA	OEA	PEA
**Cortisol**						
*r*_S_		− 0.371*	0.006	− 0.216	− 0.050	− 0.054
*p*		0.011^a^	0.979^b^	0.121^d^	0.723^d^	0.700^d^
**2-AG**						
*r*_S_			0.188	0.353**	0.166	0.255*
*p*			0.337^c^	0.006^e^	0.205^e^	0.049^e^
**AEA**						
*r*_S_				0.114	0.188	0.232
*p*				0.541^f^	0.311^f^	0.209^f^
**SEA**						
*r*_S_					0.557***	0.705***
*p*					< 0.001^g^	< 0.001^g^
**OEA**						
*r*_S_						0.884***
*p*						< 0.001^g^
*Med* (*IQR*) [pg/mg]	14.4 (9.5)	1331.6 (1257.9)	2.2 (1.4)	5265 (5123)	2161 (3867)	3078 (5061)
*Range* [pg/mg]	4.1–70.7	310.7–11,659.0	0.8–7.7	1962–38,875	815–57,619	1071–40,952
*N*	53	60	31	72	72	72

Spearman rank correlations (*r*_S_), *p < 0.050, **p < 0.010, ***p < 0.001, two-tailed. Bold-faced correlations are significant. Underlined *p*-values are significant after correction with false discovery rate. Sample sizes vary due to differing number of detected steroids and lipids in hair: *n* = ^a^46, ^b^23, ^c^28, ^d^53, ^e^60, ^f^31, ^g^72.

## Discussion

In our study, we addressed possible alterations in the interplay of cortisol as well as selected endocannabinoids and NAEs in the development of mental and physical health problems after exposure to chronic and traumatic stress^6,20^. Among our study cohort, i.e. EMS personnel, we found higher HCC in individuals with higher workload. In addition, higher hair concentrations of 2-AG were associated with more severe CM exposure and lower HCC. Higher 2-AG, SEA, OEA, and PEA hair concentrations were observed among EMS personnel who reported more exposure to CM, major life events in adulthood, or more severe subclinical depressive and physical stress symptoms.

In our sample of EMS personnel, there was a significant negative correlation between cortisol and 2-AG concentrations in hair, whereas cortisol and AEA concentrations were not correlated. Similarly, Krumbholz et al.^28^ reported negative associations between the trajectories of glucocorticoids and 2-AG as well as AEA concentrations in hair of women over the course of pregnancy. In our study, the statistical power might have been too low to detect associations of AEA concentrations in hair. AEA could be measured in 31 hair samples only due to its lower abundance in various body matrices as compared to other endocannabinoids and NAEs^20,28^. Overall, negative correlations between AEA and 2-AG with glucocorticoids in hair suit conceptual models^6,7,11,12^ proposing (i) tonic AEA activity to suppress HPA-axis activity in the absence of stress, and (ii) a glucocorticoid activity-triggered production and secretion of 2-AG to act as a turn-off signal for the HPA axis after the stress has ended. Further in line with previous findings^28,42,43^, we found SEA, PEA, and OEA concentrations highly inter-correlated, but not associated with AEA or cortisol^28^. Inconsistent correlations between the concentrations of endocannabinoids and NAEs could indicate that not all these lipids enter the hair through the same pathway^52^. It is important to note that the incorporation of glucocorticoids, endocannabinoids, and NAEs into hair is not yet sufficiently understood^10,26,28^.

### Hair cortisol concentration among EMS personnel

We observed higher HCC among EMS personnel who reported higher workload within the month prior to hair sampling. This observation suits previous evidence of higher HCC among individuals with ongoing mental and physiological stress, e.g. industrial shift workers, nursing personnel, or young parents^9,10^. Shift work-related disturbances of the sleep rhythm can trigger changes in cortisol metabolism and distribution in the body^5,53,54^. Higher HCC and correspondingly, higher diurnal cortisol secretion are typical observations among shift workers^5,53,55^. Intervention studies need to examine (i) whether elevated cortisol activity predicts stress-related mental and physical morbidity among occupational cohorts, as well as, (ii) whether preventive measures of work organisation and occupational health management can reverse elevated HCC.

We expected to find reduced HCC among EMS personnel who reported higher exposure to potentially traumatic life events. However, HCC did not correlate with their lifetime exposure to potentially traumatic event types, experienced on duty or in private life. Our finding contradicts with a widely proposed perspective that temporary increases of cortisol secretion after trauma exposure could possibly sensitise the HPA axis, causing a reduced tonic cortisol activity in the long term^34,35^. Similar to our finding, previous research also provided highly inconsistent evidence for reduced HCC among individuals with recurrent trauma exposure^36–38^. Longitudinal studies among trauma-exposed professions (e.g. EMS, fire department, military) are highly relevant in order to examine whether recurrent trauma exposure causes alterations of HCC and intra-corporeal cortisol dynamics, and also, to investigate whether it may be normalised through trauma-focused psychotherapy^37,56^. Furthermore, it is significant to explore how regulatory alterations in the endocrine stress response are represented on other biological levels, e.g. changes in receptor densities, protein translation, and gene methylation.

A trauma-related reduction of tonic cortisol activity was suggested as a risk factor for the development of PTSD symptoms after re-traumatisation^32,40,41^. A meta-analysis^9^ assembling strongly heterogeneous studies concluded on a weak negative association between PTSD symptoms and HCC. Furthering this heterogeneous body of evidence, there was no association between HCC and subclinical PTSD symptoms in our study. Moreover, in line with the meta-analysis^9^, we observed no association between the HCC and subclinical depressive symptoms among EMS personnel. Our finding further corresponds to evidence from a longitudinal study among medical students indicating no pro- or retrospective associations between HCC changes and the progression of subclinical depressive symptoms^29^. To our best knowledge, our study is the first to investigate the association between physical stress symptoms and HCC among a high-risk profession, but we did not find any significant association. Longitudinal studies among high-risk professionals are necessary to clarify whether changes in HCC present a sensitive and robust marker to objectify the risk of stress-related mental or psychosomatic health problems.

Previous research yielded inconsistent findings on altered HCC among adults with a history of CM^36,39^. In our cohort of EMS personnel, there was no correlation between HCC and the severity of experienced CM. This observation complements previous findings of null correlations in adult CM survivors^36,39^. Preliminary evidence suggested that the inconsistent findings across previous studies might be the result of gene × environment interactions in the regulation of glucocorticoid activity^39^. Future studies should therefore consider additional biological information on genes associated with the regulation of the endocrine stress response, e.g. FKBP5 or NR3C1.

### Endocannabinoid and NAE hair concentrations among EMS personnel

Our study is the first to investigate endocannabinoid and NAE concentrations in the hair of a professional cohort with frequent occupational and traumatic stress exposure. We found no consistent association between the investigated lipids and the participants’ quantitative workload which was measured based on the number of nightshifts, medical rescue operations, and routine patient transports carried out within a month prior to hair sampling. In this cohort of predominantly healthy EMS personnel, higher 2-AG, SEA, OEA, and PEA hair concentrations were linked to more severe CM experiences, whereas higher OEA and PEA concentrations in hair correlated with higher exposure to private major life events. There was no correlation observed between endocannabinoid/NAE concentrations and exposure to potentially traumatic event types on duty. Moreover, participants with more subclinical depressive and/or physical symptoms exhibited higher SEA, PEA, and OEA concentrations. When interpreting higher endocannabinoid and NAE hair concentrations as an indicator of an elevated intra-corporal activity of these lipids, our observation could refer to an increased mobilisation of 2-AG, PEA, SEA, and OEA. Activated immune cells secrete endocannabinoids and NAEs to the circulation in order to prevent further increase in immune activity and contribute to recover the immune cell activation back to baseline^14,20,43^. Thus, higher endocannabinoids and NAEs might mirror a regulatory effort, i.e. the allostatic load of the body in attempting to compensate the chronic low-grade pro-inflammatory phenotype that typically characterises individuals with a history of CM, traumatic experiences, or current depression and PTSD^15,17,18^.

Nevertheless, the direction of our findings is contrary to previous investigations of endocannabinoids and NAEs in human hair^42,43^. Higher lifetime trauma exposure correlated with lower PEA, SEA, and OEA hair concentrations among Ugandan civil war survivors with trauma-associated mental health problems^42^. And in a cohort of women in one month postpartum^43^, CM exposure was associated with higher 1-AG and lower PEA, however there was no association with SEA, AEA, and OEA concentrations in hair. In contrary to our findings, these studies reported lower endocannabinoid and NAE concentrations among individuals with psychiatric lifetime diagnosis^43^ and severe PTSD symptoms^42^. The disparity might result from the fact that our cohort was predominantly mentally and physically healthy, whereas the cohorts of previous studies comprised of war-traumatised PTSD patients^42^ as well as women with concurrent physiological alterations following pregnancy and parturition^43^.

Another possible influence on the regulation of cortisol, endocannabinoids, and NAEs might be the dietary habits and metabolic profile of EMS personnel. Enforced by working in shifts, EMS personnel tend to be on irregular/high-calorie diet as well as frequent alcohol consumption, often resulting in obesity and metabolic syndrome-related secondary diseases^5,54,57^. Previous studies found higher HCC among individuals with higher BMI^5,9^. Moreover, there is consistent evidence that anticipation and consumption of high-calorie food causes an increase in 2-AG, AEA, and higher intestinal OEA concentrations in blood^13,58^. Subsequently, AEA and 2-AG increase sensitivity to the sweet taste and thus influence the preference for high-calorie food^13,58^. OEA also influences the feeling of satiety and the storage of energy by de novo lipogenesis, and, hence, play a role in development of obesity^13,58^. However, in our study, cortisol, endocannabinoid, and NAE hair concentrations did not correlate with the BMI of EMS personnel. Moreover, individuals with obesity-related diseases such as type-II diabetes or hypertension were excluded since these conditions may bias the HCC^9,44^. Future longitudinal studies are needed to investigate whether changes in sleep quality, lifestyle, and nutrition behaviour resulting from adverse working conditions (e.g. shift and night work) translate into negative health outcomes by altering diurnal cortisol, endocannabinoid, and NAE dynamics^5^.

### Strengths and limitations

This study is the first to co-investigate cortisol, endocannabinoids, and NAEs in the hair of chronically stressed and frequently trauma-exposed professionals. We provided a comprehensive overview of steroid and lipid concentrations and their associations with several types of private and duty-related stress. In addition, we controlled various covariates that could systematically influence HCC^9,28,44,50^. The relatively small sample size limits the reliability and generality of our results. Specifically, the results regarding AEA are to be interpreted with caution, as the lipid could be measured in 31 hair samples only due to its lower abundance in various body matrices as compared to other endocannabinoids and NAEs^20,28^. In future studies, one way to reduce the number of unsuccessful measurements of target substances could be to standardise the amount of hair supplied to the laboratory analyses. Moreover, the study’s cross-sectional, retrospective nature does not allow causal or longitudinal conclusions. In addition, using hair concentrations as a marker of long-term endocannabinoid and NAE activity in the body needs to be validated via endocannabinoid and NAE profiles in blood, saliva, and urine. Moreover, compared to cortisol, relatively little is known about the import kinetics of endocannabinoids and NAEs from the hair follicle into the hair shaft as well as these substances’ temporal deposition in hair^52^. To date, interpreting hair-based biomarkers remains challenging, as the physiological mechanisms of glucocorticoid, endocannabinoid, and NAE incorporation into hair have not yet been sufficiently undersetood^10,26,28^. Therefore, future research is needed to better characterise the deposition and abundance of lipids and steroids in hair.

## Conclusions

This was the first study which simultaneously analysed hair-based markers of alterations in the co-regulation of the HPA axis and the endocannabinoid/NAE system in a stress and trauma-exposed cohort. Higher cortisol concentrations were found in hair of EMS personnel with higher quantitative workload which corroborates the established meta-analytical perspective of hair cortisol as a marker of ongoing stress and shift work. Advancing existing evidence, we found higher endocannabinoid and NAE levels in EMS personnel who reported more exposure to CM, major life events in adulthood, or more severe subclinical depressive and physical stress symptoms. This finding points towards a possible role of endocannabinoids and NAEs in translating the CM-related lifetime vulnerability for trauma-related mental and physical health problems. There is a need for longitudinal studies among recurrently stressed cohorts in order to examine the interplay of glucocorticoid, endocannabinoid, NAE, and inflammatory dynamics across different biological matrices (e.g. hair, blood, urine, saliva). For the same, future studies need to recruit larger and more diverse study cohorts for comparing occupations with various stress and trauma exposure. It is also relevant to investigate whether tracing alterations of biomolecular markers of work-related stress enables predicting the onset of mental or somatic health problems.

## Supplementary Information

Supplementary Tables.

## Data Availability

The data generated and analysed during the current study are not publicly available, as we do not have the consent of the ethics committee or our participants to grant access to or insight into the collected data. As a result, the data may not be published in public repositories and may not be made accessible to any third parties outside the research project.
