# Supplementary material for: Associating Emergency Medical Services personnel’s workload, trauma exposure, and health with the cortisol, endocannabinoid, and N-acylethanolamine concentrations in their hair
Source: Sci Rep. 2020 Dec 29;10:22403. doi: 10.1038/s41598-020-79859-x (PMC7772331; doi:10.1038/s41598-020-79859-x)
Supplement: Supplementary file 1 — Supplementary Tables. [file 41598_2020_79859_MOESM1_ESM.docx]

Associating Emergency Medical Services personnel’s workload, trauma exposure, and health with the cortisol, endocannabinoid, and *N*-acylethanolamine concentrations in their hair

**— Supplementary Material —**

**Authors**: Alexander Behnke^1^*, Alexander Karabatsiakis^1,2^, Aniko Krumbholz^3^, Sarah Karrasch^1^, Gustav Schelling^4^, Iris-Tatjana Kolassa^1^, Roberto Rojas^5^

^1^ Clinical & Biological Psychology, Institute of Psychology and Education, Ulm University, DE-89081 Ulm, Germany

^2^ Institute of Psychology, University of Innsbruck, AT-6020 Innsbruck, Austria

^3^ Institute of Doping Analysis and Sports Biochemistry Dresden, DE-01731 Kreischa, Germany

^4^ Department of Anesthesiology, Ludwig Maximilians University, DE-82131 Munich, Germany

^5^ University Psychotherapeutic Outpatient Clinic, Institute of Psychology and Education, Ulm University, DE-89073 Ulm, Germany

* Correspondence should be addressed to alexander.behnke@uni-ulm.de

Supplementary Table S1. Results of Mann-Whitney *U*-Test on differences between men and women

|  | **Cortisol** | **2-AG** | **AEA** | **SEA** | **OEA** | **PEA** |
| --- | --- | --- | --- | --- | --- | --- |
| **women** *Med* (*IQR*) in pg/mg | 14.8 (7.6) | 1708.3 (1237.7) | 2.7 (2.9) | 6026 (9842) | 4538 (7509) | 4562 (10018) |
| **men** *Med* (*IQR*) in pg/mg | 13.0 (5.9) | 1345.5 (1563.2) | 2.2 (1.2) | 5020 (3942) | 1893 (1867) | 2614 (3435) |
| ***N* (n_f_:n_m_)** | 44 (20:24) | 52 (26:29) | 28 (11:17) | 62 (27:35) | 62 (27:35) | 62 (27:35) |
| ***U*** | 179.00 | 264.00 | 83.00 | 344.00 | 239.00 | 281.00 |
| ***z*** | -1.44 | -1.28 | -0.49 | -1.82 | -3.32 | -2.72 |
| ***p*** | .150 | .200 | .621 | .068 | **<.001** | **.007** |

Supplementary Table S2. Results of Mann-Whitney *U*-Test on differences due to hair treatment

|  | **Cortisol** | **2-AG** | **AEA** | **SEA** | **OEA** | **PEA** |
| --- | --- | --- | --- | --- | --- | --- |
| **no** *Med* (*IQR*) in pg/mg | 14.2 (9.2) | 1265.8 (1334.3) | 2.2 (1.3) | 5094 (4063) | 2089 (3719) | 2879 (4261) |
| **yes** *Med* (*IQR*) in pg/mg | 14.4 (10.0) | 1927.7 (1280.4) | 3.0 (2.9) | 9468 (11957) | 7128 (9061) | 9835 (11007) |
| ***N* (n_no_:n_yes_)** | 53 (46:7) | 59 (52:7) | 30 (24:6) | 71 (63:8) | 71 (63:8) | 71 (63:8) |
| ***U*** | 158.00 | 118.00 | 63.00 | 149.00 | 127.00 | 136.00 |
| ***z*** | -0.08 | -1.50 | -0.47 | -1.87 | -2.27 | -2.11 |
| ***p*** | .937 | .134 | .641 | .061 | **.023** | **.035** |

*Note*: Bleaching, dyeing, tinting, and permanent waving in the last 6 weeks before hair sample collection were considered as a hair treatment (one missing value).

Supplementary Table S3. Results of Mann-Whitney *U*-Test on differences due to natural hair colour

|  | **Cortisol** | **2-AG** | **AEA** | **SEA** | **OEA** | **PEA** |
| --- | --- | --- | --- | --- | --- | --- |
| **red to dark blond** *Med* (*IQR*) in pg/mg | 13.4 (7.6) | 1425.7 (1469.4) | 1.96 (1.5) | 5057 (5483) | 2436 (4747) | 3172 (5809) |
| **brunette to black** *Med* (*IQR*) in pg/mg | 14.4 (11.4) | 1177.1  (1240.7) | 2.49 (1.2) | 5483 (4590) | 2148 (3585) | 3146 (5175) |
| ***N* (n_blo_:n_bru_)** | 53 (24:29) | 59 (27:32) | 30 (14:16) | 71 (30:41) | 71 (30:41) | 71 (30:41) |
| ***U*** | 343.00 | 348.00 | 85.00 | 595.00 | 609.00 | 597.00 |
| ***z*** | -0.09 | -1.28 | -1.12 | -0.23 | -0.07 | -0.21 |
| ***p*** | .929 | .201 | .262 | .816 | .944 | .834 |

*Note*: Red and blond hair and brunette and black were each summarised as only a small percentage of participants had red (*n* = 2) or black (*n* = 3) as natural hair colour. Participants with grey hair were categorised according to their initial natural hair colour (one missing value).

Supplementary Table S4. Results of Mann-Whitney *U*-Test on differences due to self-reported acute inflammatory conditions

|  | **Cortisol** | **2-AG** | **AEA** | **SEA** | **OEA** | **PEA** |
| --- | --- | --- | --- | --- | --- | --- |
| **no** *Med* (*IQR*) in pg/mg | 14.4 (9.2) | 1425.7 (1185.4) | 2.7 (1.5) | 5703 (5894) | 2148 (5060) | 3336 (6143) |
| **yes** *Med* (*IQR*) in pg/mg | 13.6 (9.4) | 1102.6 (1052.2) | 2.2 (1.3) | 4678 (4711) | 2190 (3581) | 2682 (3898) |
| ***N* (*n*_no_:*n*_yes_)** | 53 (29:24) | 60 (37:23) | 31 (21:10) | 72 (43:29) | 72 (43:29) | 72 (43:29) |
| ***U*** | 331.00 | 366.50 | 89.00 | 518.00 | 583.50 | 579.00 |
| ***z*** | -0.30 | -0.90 | -0.68 | -1.21 | -0.46 | -0.51 |
| ***p*** | .761 | .370 | .499 | .226 | .646 | .609 |

*Note*: The most common acute inflammatory conditions reported at the time of hair sample collection or in the four weeks prior to hair sample collection were common colds, flu, influenza, angina, and cystitis.

Supplementary Table S5. Results of Mann-Whitney *U*-Test on differences due to experiencing major stress events within four weeks before study participation

|  | **Cortisol** | **2-AG** | **AEA** | **SEA** | **OEA** | **PEA** |
| --- | --- | --- | --- | --- | --- | --- |
| **no** *Med* (*IQR*) in pg/mg | 14.1 (5.9) | 1391.7 (1410.7) | 2.5 (1.2) | 5061 (6772) | 2224 (3528) | 3420 (4754) |
| **yes** *Med* (*IQR*) in pg/mg | 12.7 (3.0) | 1380.3 (1385.7) | 1.9 (2.3) | 6320 (5018) | 3222 (5837) | 3344 (7279) |
| ***N* (*n*_no_:*n*_yes_)** | 41 (31:10) | 49 (33:16) | 26 (18:8) | 59 (42:17) | 59 (42:17) | 59 (42:17) |
| ***U*** | 118.00 | 215.00 | 53.00 | 308.00 | 278.00 | 327.00 |
| ***z*** | -1.12 | -1.05 | -1.06 | -0.82 | -1.32 | -0.50 |
| ***p*** | .261 | .296 | .291 | .412 | .186 | .616 |

*Note*: Participants characterised the type of experienced stressful events on a free text item and rated the event-related subjective stress on a 4-point Likert scale from 0 (“not stressful at all/no event”) to 3 (“very stressful”). Events rated as at least 2 (“stressful”) were considered major stress event (two missing values).

Supplementary Table S6. Bivariate Spearman correlations (*r*_S_) between cortisol and endocannabinoid concentrations in hair and potential influencing factors

|  |  | Cortisol | 2-AG | AEA | SEA | OEA | PEA |
| --- | --- | --- | --- | --- | --- | --- | --- |
| Age | *r*_S_ | -.201 | -.273 | -.045 | -.195 | -.201 | **-.274*** |
|  | *p* | .191 | .050 | .818 | .129 | .118 | .031 |
|  | *N* | 44 | 52 | 28 | 62 | 62 | 62 |
| EMS work experience | *r*_S_ | -.137 | -.112 | -.128 | -.020 | -.005 | -.050 |
|  | *p* | .376 | .430 | .515 | .880 | .967 | .701 |
|  | *N* | 44 | 52 | 28 | 62 | 62 | 62 |
| Body Mass Index | *r*_S_ | -.249 | -.067 | .377 | .381 | .321 | .216 |
|  | *p* | .353 | .768 | .318 | .066 | .126 | .311 |
|  | *N* | 16 | 22 | 9 | 24 | 24 | 24 |
| Sports exercises (hours per week) | *r*_S_ | .014 | .146 | .042 | .067 | .009 | .111 |
|  | *p* | .923 | .268 | .825 | .581 | .941 | .355 |
|  | *N* | 53 | 59 | 30 | 71 | 71 | 71 |
| Frequency of hair washing per week | *r*_S_ | -.044 | -.180 | -.186 | .009 | -.116 | -.076 |
|  | *p* | .756 | .171 | .324 | .938 | .337 | .528 |
|  | *N* | 53 | 59 | 30 | 71 | 71 | 71 |

*Note*: * *p* < .050, ** *p* < .010, *** *p* < .001, two-tailed.

Supplementary Table S7. Bivariate Spearman rank correlations (*r*_S_) between cortisol and endocannabinoid concentrations in hair and different stress types and symptoms

|  |  | Cortisol | 2-AG | AEA | SEA | OEA | PEA |
| --- | --- | --- | --- | --- | --- | --- | --- |
| Quantitative workload | *r*_S_ | **.419**** | -.122 | .202 | -.125 | .097 | .041 |
|  | *p* | .005^a^ | .387^e^ | .303^i^ | .332^l^ | .454^l^ | .755^l^ |
| Childhood maltreatment exposure (MACE-20) | *r*_S_ | -.023 | **.290*** | .086 | .213 | **.286*** | .235 |
|  | *p* | .886^b^ | .039^f^ | .669^j^ | .099^m^ | .025^m^ | .069^m^ |
| Number of experienced major life events (private life) (LEC-5) | *r*_S_ | -.169 | -.018 | .133 | .091 | **.284*** | .232 |
|  | *p* | .274^a^ | .900^e^ | .499^i^ | .480^l^ | .025^l^ | .069^l^ |
| Number of experienced potentially traumatic mission aspects (RESQ-CE) | *r*_S_ | -.129 | -.052 | .091 | -.004 | .097 | .008 |
|  | *p* | .408^b^ | .716^f^ | .652^j^ | .978^m^ | .455^m^ | .952^m^ |
| Severity of posttraumatic stress symptoms (PCL-5) | *r*_S_ | -.027 | .097 | -.008 | .165 | .134 | .080 |
|  | *p* | .864^c^ | .503^g^ | .969^k^ | .207^n^ | .309^n^ | .542^n^ |
| Severity of physical symptoms (PHQ-15) | *r*_S_ | -.249 | -.087 | -.063 | .150 | **.398**** | .238 |
|  | *p* | .116^d^ | .551^h^ | .760^k^ | .256^o^ | .002^o^ | .070^o^ |
| Severity of depressive symptoms (PHQ-9) | *r*_S_ | -.208 | -.103 | -.050 | **.301*** | .212 | .103 |
|  | *p* | .192^d^ | .479^h^ | .809^k^ | .020^o^ | .107^o^ | .437^o^ |

*Note*: * *p* < .050, ** *p* < .010, *** *p* < .001, two-tailed. Sample sizes differ depending on available data: *n* = ^a^ 44, ^b^ 43, ^c^ 42, ^d^ 41, ^e^ 52, ^f^ 51, ^g^ 50, ^h^ 49, ^i^ 28, ^j^ 27, ^k^ 26, ^l^ 62, ^m^ 61, ^n^ 60, ^o^ 59.
